# Maternal near miss and mortality in a tertiary care hospital in Rwanda

**DOI:** 10.1186/s12884-015-0619-8

**Published:** 2015-09-03

**Authors:** Stephen Rulisa, Immaculee Umuziranenge, Maria Small, Jos van Roosmalen

**Affiliations:** Department of Obstetrics and Gynecology, University of Rwanda, Kigali, Rwanda; Department of Obstetrics, Leiden University Medical Center, Leiden, The Netherlands; Department of Obstetrics and Gynecology, Duke University, Durham, NC USA; Athena Institute, VU University Amsterdam, Amsterdam, The Netherlands

**Keywords:** Maternal mortality, Maternal near miss, Peritonitis, Obstetric infection, Cesarean section, Low-income country

## Abstract

**Background:**

To determine the prevalence and factors associated with severe (‘near miss’) maternal morbidity and mortality in the University Teaching Hospital of Kigali – Rwanda.

**Methods:**

We performed a cross sectional study of all women admitted to the tertiary care University Hospital in Kigali with severe “near miss” maternal morbidity and mortality during a one year period using the WHO criteria for ‘near miss’ maternal mortality. We assessed maternal demographic characteristics and disease processes associated with severe obstetric morbidity and mortality.

**Results:**

The prevalence of severe maternal outcomes was 11 per 1000 live births. The maternal near miss ratio was 8 per 1000 live births. The majority of severe obstetric morbidity and mortalities resulted from: sepsis/peritonitis (30.2 %)--primarily following caesarean deliveries, hypertensive disease (28.6 %), and hemorrhage (19.3 %). Majority of our patients were found to be of lower socioeconomic status, refered from district hospitals to the tertiary care center, and resided in the eastern part of the country.

**Conclusion:**

The main causes associated with MNH were peritonitis, hypertensive disorders and bleeding. The high prevalence of peritonitis may reflect suboptimal intraoperative and intrapartum management of high-risk patients at district hospitals. Direct causes of severe maternal outcome are still the most prevalent.

The study identified opportunities for improvement in clinical care to reduce potentially these adverse outcomes.

## Background

Maternal mortality remains a major challenge to health systems worldwide [1]. The focus on maternal mortality was sharpened when reduction of maternal mortality became one of eight Millennium Development Goals [MDG] [1]. The target for MDG 5 is to reduce the maternal mortality ratio (MMR) by three-quarters from 1990 to 2015 [1]. However, achieving progress towards that goal has proven challenging.

Sub-Saharan Africa has some of the highest maternal mortality ratios (MMR) in the world, often double the globally estimated MMR of 400 per 100,000 live births. Because of high fertility and poor access to health care, African women face an extremely high lifetime risk of maternal death; 1 in 20 for sub-Saharan African women compared with 1 in 2400 for women in the USA [1]. The HIV epidemic in sub-Saharan Africa is considered partially culpable for the slow progress in MMR reduction for sub-Saharan Africa [2]. Despite these challenges, several demographic and social factors associated with maternal mortality have improved in many countries. Reductions in total fertility, increases in income per head, increase in maternal education and social stability are factors enabling many countries to reduce maternal death [1].

In Rwanda, significant investments and advancements have been made to improve the infrastructure of primary health centers, educational opportunities, medical teaching programs and health care access through community health insurance [3, 4].

The average MMR during the 5 years before 2000 stood at 1071 per 100,000 live births and decreased to 750 for the 5 years preceding 2005 [1]. It further decreased to 476 for the 5 years preceding 2010 [3]. These improvements have occurred largely due to political stability and policy change focused on health care improvement [4]. In 2010, the leading cause of maternal mortality was from bleeding complications. Sepsis was the second leading cause of maternal death. The average cesarean section rate for all hospitals increased from 35 % in 2010 to 45 % in 2011 [5]. Access to cesarean deliveries is associated with improved maternal and perinatal outcomes in countries with poor access to obstetric care, however, high cesarean rates may contribute to increased maternal morbidity and mortality [6].

Mantel et al. describe the course from uncomplicated pregnancy to maternal death, as a continuum and surveillance of severe morbidities may also add to maternal mortality reduction efforts [7]. Measures of severe maternal morbidity or ’near miss’ may enhance surveillance of adverse events. According to Burchett, “near misses are more common than death, allowing more statistical precision and smaller sample sizes” [8].

Over the last decade, Rwanda has made great progress in maternal mortality reduction, however, further progress is needed. This study evaluated the prevalence, and causes of maternal mortality and ‘near miss’ maternal morbidity in the University teaching hospital of Kigali (CHUK). We assessed the factors associated with severe morbidity and mortality to determine the potential preventability of these outcomes.

## Methods

We evaluated the prevalence and risk factors associated with severe maternal morbidity (‘near miss’) and mortality in the CHUK hospital in Kigali-Rwanda. CHUK is the largest public hospital in Rwanda and serves as one of the primary teaching and tertiary care referral centers for the country.

Routinely, single dose prophylactic antibiotic therapy is given for all cesarean deliveries.

The study protocol was reviewed and approved by the institutional review board of University Teaching Hospital of Kigali and by the research committee of Leiden University Medical Center in the Netherlands.

This cross sectional study included all patients admitted to the CHUK obstetrics service with severe maternal morbidity or mortality. The study period was from October 2011 until October 2012. All patient signed a written informed consent

We used the WHO multi-country survey on maternal and newborn health surveillance system to evaluate factors associated with severe maternal morbidity and mortality [9]. Patients were identified through review of the obstetrics &gynecology admissions log and through provider identification of eligible patients. Data were collected from the time of admission or at a later moment during the patient’s stay at the hospital depending on the condition of the patient. The WHO surveillance form was used to capture data for every woman who presented with severe maternal morbidity or who died during or after admission.

In addition to medical record review, a subset of patients more history was obtained from them to obtain a clearer picture of the factors leading to their adverse outcomes. Each participant interviewed received verbal and written explanation of the study in Kinyarwanda or English before consent was obtained.

Initially we wanted to use the WHO maternal near-miss criteria [9] to choose cases we considered as severe maternal morbidities (Table 1). In most cases, it was impossible to meet the full WHO criteria because most of the laboratory tests used to define those events, were not performed at CHUK hospital. So additionally, patients were also included if they had severe maternal complications at baseline or received critical interventions or intensive care unit admission according to the WHO guidelines. We included hemorrhage cases if 5 or more units of blood were given. One, trained researcher identified participants (UI) and the principal investigator verified suitability for study inclusion (SR).

**Table 1:** Maternal Demographic Characteristics of women with severe maternal morbidity in Rwanda Number (percent of cohort)

Age	
> 20	13(6.8)
20–35	149(77.6)
36–45	30(15.6)
Socioeconomic Status	
Low	149(77.6)
Middle	37(19.2)
High	0
Unknown	6(3.1)
Body Mass Index	
< 18.5	5(2.6)
18.5–24.9	154(80.2)
25–29.9 (overweight)	28(14.6)
30–35(obese)	5(2.6)
Geographic Region	
Kigali	60(31.2)
Eastern Province	73(38)
Southern Province	27(14.1)
Northern Province	21(10.9)
Western Province	11(5.7)
Marital Status	
Married/Cohabiting	44(22.9)
Single/Divorced/Widowed	148(77.1)

The denominator included all patients who delivered live babies at CHUK hospital during the study period. Because CHUK hospital is the largest, tertiary care referral hospital in Rwanda, the patient population represents women from the entire country.

We applied the WHO definitions of severe maternal outcome and maternal near miss ratios in order to assess the need for medical care resource allocation in a facility or area [9]. The severe maternal outcome (SMO) ratio is defined as the number of women with life threatening conditions (maternal death + maternal near miss cases) per 1000 live births in the study period and the Maternal Near Miss Mortality Ratio (MNM: 1MD) as the ratio between near misses cases and deaths. Higher ratios are associated with a higher quality of care [9]. The mortality index (MI) is defined as the number of maternal deaths divided by the number of near miss cases + maternal deaths. The higher the index, the lower the quality of care [9].

Once data collection period was over the information was entered and descriptive statistics (percentiles) were analyzed using SPSS v16.0.

## Results

We identified 192 cases of severe near miss maternal morbidity and mortality during the study period. In the same period, 1739 live births occurred. The overall cesarean delivery rate for CHUK was 45 % during the study period. The severe maternal outcome ratio was 11 per 1000 live births. The maternal near miss ratio was 2.8 and the maternal mortality index was 0.26 for our cohort.

Most women with maternal morbidity were in the childbearing years 20–35 (78 %). Women above age 35 comprised 15 % of the cohort. The majority of women were either married or cohabiting (83.3 %). Socioeconomic status was identified as low (as per their insurance category) in 78 %, and the majority (80 %) had a normal range body mass index (BMI 18.5–24.9). Most patients resided in either Kigali (31 %) or the Eastern province (38 %) (Table 1).

The most common causes of severe maternal morbidity resulted from peritonitis (30.2 %), hypertensive disease (28.6 %), hemorrhage (19.3 %) and cardiomyopathy (5.2 %) (Table 2).

**Table 2:** Primary causes of Severe Maternal Outcome (SMO) and Mortality (M) in Rwanda

Condition	SMM and M, number (%)	Maternal deaths
Peritonitis	58(30)	16
Hemorrhage	45(18.7)	10
Hypertensive disease	55(28.6)	9
Cancer	5(1.5)	4
-pancreatic (1)		
-breast cancer (2)		
-leukemia (1)		
Cardiomyopathy	7(3.6)	3
Malaria	6(3)	2
Pulmonary Embolism	2 (2.2)	0
Pulmonary Disease	1 (1.1)	1
Diabetes	2(2.2)	
-severe hypoglycemia,		
-diabetic coma		
Hepatic Disease	2(2.2)	0
-cirrhosis		
-hepatic abscess		
Neurologic	2(2.2)	
-stroke postpartum		
-coma of unknown etiology)		
Anesthetic complications	2(2.2)	
Epilepsy	1 (1.1)	0
Other*	7(3.6)	5
Total number of patients experiencing severe maternal morbidity + mortality^a^	192	50

^a^the total number of patients =192, although some patients experienced more than one severe morbidity

Other causes of severe maternal morbidity and mortality included: motor vehicle accident (1), Mallory Weiss syndrome (1), tonsillitis treated with traditional medicine resulting in sepsis and death (1), hemothorax(1) anaphylactic shock(1)

Of all the women with severe obstetric morbidity, 36 % were nulliparous. Seventeen percent had prior cesarean sections compared to 78 % without prior cesarean sections. For 5.2 % of cases it was unclear whether they had prior cesarean sections because it was not clearly indicated in their transfer note from the district. Of the women who delivered, 58 % had a cesarean section compared to 26 % who had a vaginal delivery. The majority of women (90 %) delivered in a hospital or health center. Fifty-five percent of women had term pregnancies; 45 % had preterm births or abortions. Only two women smoked in the cohort. Ninety eight percent of women had one antenatal care visit and only 35 % had 4 or more visits. The majority of patients (90 %) in the cohort were referred to CHUK from district hospitals.

Fifty pregnancy related deaths occurred during the study period, resulting in an overall case fatality rate of 26 %. In more than 50 % of the cases, death occurred in the postpartum period, 28 % in early pregnancy and 22 % antepartum. Maternal deaths resulted from peritonitis/sepsis, hemorrhage, cardiomyopathy, and breast cancer, respectively. Hypertensive disorders (primarily severe preeclampsia/eclampsia) were the second leading cause of severe maternal morbidity; one maternal death resulted from eclampsia.

A total of 75 cases of severe maternal morbidities and mortalities resulted from infections, giving a prevalence of 43 per 1000 live births. Peritonitis complicated 58 of these cases, giving a prevalence of 30 per 1000 live births. In 17 patients, infection was a secondary diagnosis (not the primary indication for admission). There were 16 maternal mortalities due to peritonitis, giving a case fatality rate of 28 %. Fifty percent (29 patients) patients with peritonitis were primiparas who underwent cesarean deliveries. Half of the patients with peritonitis were transferred from a single region—the Eastern province.

A total of 45 cases of severe obstetric hemorrhage were reported, giving a prevalence of major obstetric hemorrhage of 26 per 1000 live births (Table 3). Ten women died secondary to obstetric hemorrhage, resulting in a case fatality rate of 22 %. In all cases blood products were given; ten patients required hysterectomy. One patient, experienced obstetric hemorrhage and died after declining blood transfusion.

A total of 55 patients experienced complications from hypertensive disease, giving a prevalence of 32 per 1000 live births. The majority of patients experienced severe preeclampsia (20) and eclampsia (33). Eight patients were diagnosed with HELLP syndrome. There were 9 deaths due to hypertensive disease. Eight maternal deaths resulted from eclampsia and 1 from severe preeclampsia giving a case fatality rate of 16 %.

HIV complicated nine pregnancies (4.7 %) and was not a primary cause of severe maternal morbidity or mortality.

Peripartum cardiomyopathy occurred in 7 patients, giving a prevalence of 4 per 1000 deliveries. Three of these patients died resulting in a case fatality rate of 42 %.

There were six cases of malaria and other related conditions, giving a prevalence of 3 per 1000 deliveries.

**Table 3:** Major obstetric hemorrhage associated with severe maternal outcomes (SMO) in Rwanda

Number (% of severe morbidity/mortality cases)^a^	
No Hemorrhage	147(76)
Postpartum Hemorrhage	23 (51)
Uterine Rupture	10(5)
Ruptured Ectopic	5(2.6)
Placenta previa, accrete/increta/percreta	3(6.6)
Abortion Related	3(1.6)
Other (motor vehicle accident)	1 (2)

^a^some patients experienced more than one severe morbidity; 36 (18.7 %) of patients experienced hemorrhage as the primary diagnosis

## Discussion

The majority of severe obstetric morbidity and mortalities resulted from sepsis/peritonitis This was a preliminary study to examine severe, ‘near miss’ maternal morbidity and mortality in the largest public teaching hospital in Rwanda, CHUK. The high prevalence of maternal morbidity and mortality likely reflects the tertiary nature of this hospital that accepts referrals from a large catchment area and many of the patients who died or experienced severe maternal morbidity/mortality died soon after transfer. These findings may not reflect outcomes at the level of the district hospital or community health center.

The high prevalence of peritonitis and corresponding high case fatality rate of 28 % is concerning. This study was unable to examine the root causes for high peritonitis referrals and subsequent severe morbidity/mortality. These findings raise several areas of concern. They may reflect a lack of experience, since doctors working in District Hospitals may be recent medical school graduates who have not completed residency programs [10]. They may also reflect unsterile techniques. Other concerns relate to proper and sufficient materials: do district hospitals use appropriate antibiotics and sterilization procedures to reduce risks [11]? As a result of these findings, providers from CHUK are visiting district hospitals and evaluating practices in order to determine problems or underlying causes for these severe, potentially preventable outcomes. The cesarean section rate for this cohort (58 %) was higher than the general rate for CHUK (45 %). WHO recommends cesarean section rates of 15 % and identifies higher rates as both potentially harmful and costly to mothers and health care systems [6].

This study also demonstrates a high prevalence of hypertensive disease related severe morbidity. These findings may reflect a high prevalence of undiagnosed chronic hypertension among women of reproductive age. In addition, low dose aspirin is recommended by WHO as a for patients at high risk for preeclampsia [12]. Education about preeclampsia/eclampsia prevention and danger signs can be heightened for both patients and providers. This type of public health initiative may reduce the severity of hypertensive disorders in pregnancy through early recognition and management. Despite the high morbidity associated with severe preeclampsia, maternal mortality death from these conditions was low in our population (Table 4).

**Table 4:** The WHO maternal near-miss criteria^a^

Identification criteria	
Cardiovascular dysfunction	• Shock
	• Use of continuous vasoactive drugs
	• Cardiac arrest
	• Cardio-pulmonary resuscitation
	• Severe hypoperfusion (lactate > 5 mmol/L or > 45 mg/dL)
	• Severe acidosis (pH < 7.1)
Respiratory dysfunction	• Acute cyanosis
	• Gasping
	• Severe tachypnea (respiratory rate > 40 bpm)
	• Severe bradypnea (respiratory rate < 6 bpm)
	• Severe hypoxemia (PAO2/FiO2 < 200 or O2 saturation < 90 % for ≥60 min)
	• Intubation and ventilation not related to anesthesia
Renal dysfunction	• Oliguria non responsive to fluids/diuretics
	• Dialysis for acute renal failure
	• Severe acute azotemia (creatinine ≥ 300 umol/ml or ≥ 3.5 mg/dL)
Coagulation/hematologic dysfunction	• Failure to form clots
	• Massive transfusion of blood or red cells (≥5 units)
	• Severe acute thrombocytopenia (<50,000 platelets/ml)
Hepatic dysfunction	• Jaundice in the presence of pre-eclampsia
	• Severe acute hyperbilirubinemia (bilirubin > 100 umol/L or > 6.0 mg/dL)
Neurologic dysfunction	• Prolonged unconsciousness (lasting ≥ 12 hours)/coma (including metabolic coma)
	• Stroke
	• Status epilepticus/uncontrollable fits
	• Total paralysis
Uterine dysfunction	• Hemorrhage or infection leading to hysterectomy

We observed a very high prevalence of peripartum cardiomyopathy (PPCM). This condition was diagnosed clinically and confirmed by echocardiogram. Our findings are comparable to studies from Haiti [13, 14]. Most women become aware of their symptoms during their second or third pregnancy, when the heart is unable to tolerate the pregnancy-related volume overload. This disease may again represent an opportunity for preconception counseling and family planning for women with previous pregnancies complicated by PPCM [15].

Obstetric hemorrhage cases were among the leading causes of severe obstetric morbidity and mortality in our population. However, hemorrhage was not the leading cause of maternal death. This finding may represent education and aggressive, standard management of obstetric hemorrhage. In Rwanda care providers, in tertiary and district hospitals, are well trained in the prevention and management of obstetric hemorrhage [16, 17]. These outcomes can be further improved, but peritonitis, hypertensive disorders and cardiomyopathy were the most common causes of severe morbidity and mortality in our study. These conditions require additional attention and focus to further reduce severe maternal morbidity and mortality.

As many studies in Rwanda demonstrate, malaria and concomitant poor obstetric outcomes have radically decreased [18–21]. This reality is also reflected by the few cases of maternal morbidity caused by malaria in our study [18, 20]. The Eastern province remains highly affected by malaria; almost all of the patients in this study resided in the Eastern province.

The fact that most patients with maternal morbidity/mortality seen in CHUK came from the eastern province can probably be explained by the fact that CHUK is the nearest tertiary care hospital to this province. Fewer transfers originate from the Western province, largely because those patients are often transferred to the University Teaching hospital of Butare (CHUB). In addition, the transfer system in the eastern province is better established than in other regions. Further work is needed to elucidate reasons for the high numbers of severe morbidities and mortalities originating from this region of the country.

Several studies have shown that even in resource limited settings maternal morbidity and mortality can be significantly reduced by improving maternal health care through strategic interventions after confidential audits [22–26]. Similar audits may help to further reduce the prevalence of maternal morbidity and mortality in Rwanda. This type of audit will be initiated at CHUK. In the cases of maternal morbidity, there is not enough critical assessment of the course that led to the severe conditions, we are still more focused on treating the condition instead of looking further on how to avoid it the next time. The subsequent audits of severe maternal morbidity and mortality will attempt to address the root causes for these adverse outcomes. Avoidance of these outcomes will necessitate not only focus on tertiary care and management, but avoidance of conditions— including social and structural—that contribute to these outcomes [8].

The primary limitation of this study is that we did not record the individual characteristics of all maternities without severe maternal morbidity during the study period. Therefore, we could not appropriately adjust relative risks for confounding variables. Furthermore, despite our efforts we cannot guarantee the completeness of our data especially because we had only one person collecting the data. Patients admitted to other wards (e.g. trauma surgery) were generally captured, but could have died without notification of the obstetric team. Therefore, our reported prevalence represent a minimum level of severe maternal morbidity and mortality.

Most patients with severe morbidity or mortality were transferred from District Hospitals. We had limited information about the use of induction or operative vaginal delivery with forceps/ventouse or the general maternal health status prior to transfer. The indications for cesarean section in patients transferred to CHUK were not clearly identified. This information void related to cesarean section indications is not unique to Rwanda and is a known barrier to the appropriate review and potential reduction of unnecessary cesarean deliveries [27]. Given the high rates of cesarean deliveries in this cohort, one of the best methods to reduce peritonitis related morbidity and mortality would be to reduce cesarean sections. Providers at CHUK are currently investigating ways to reduce the cesarean section rates in the tertiary care center and key district hospitals.

This study gives a small but important picture of the problems encountered in the largest public tertiary care center in Rwanda. There is obviously need for a larger and more detailed study to investigate the exact causes of post cesarean peritonitis and to closely review indications for primary cesarean deliveries.

Improvement of the quality of obstetric care is essential to reduce severe maternal morbidity in Rwanda. Providers from CHUK instituted visitations to district hospitals in order to provide educational support regarding best practices and to evaluate potential reasons for the high post cesarean section peritonitis rates. CHUK has initiated maternal morbidity and mortality surveillance using the WHO guidelines. The goal of these confidential audits will be to determine root causes of severe morbidity and mortality to reduce these outcomes [26].

Our findings were comparable to work from Tanzania that demonstrated a large proportion of maternal near misses and mortalities resulting from caesarean section complications [28].

## Conclusion

In our study, severe maternal morbidity complicated at least 10.4 % of all pregnancies and yielded a near miss maternal mortality ratio of 3.5 per 1000 deliveries. As the primary, national, tertiary care centre, CHUK, receives many of the most severe cases of maternal morbidity. Therefore, we believe these findings can be extrapolated to the rest of the country.

Audits of severe maternal morbidity at the district and teaching hospital should be encouraged in order to improve the quality of obstetric care and decrease the prevalence of severe maternal morbidity and mortality in Rwanda. Audits of caesarean delivery indications and practices may help reduce these risks since they would identify the exact cause. Subsequent training may help to eliminate these causes.

There is need for more research on severe maternal morbidity to validate these findings, but this should not prevent physicians from using these results to make changes in the system.
